# Tamoxifen Exerts Anticancer Effects on Pituitary Adenoma Progression via Inducing Cell Apoptosis and Inhibiting Cell Migration

**DOI:** 10.3390/ijms23052664

**Published:** 2022-02-28

**Authors:** Tingting Lv, Zirui Zhang, Haoying Yu, Shuyue Ren, Jingrong Wang, Shang Li, Lan Sun

**Affiliations:** 1 State Key Laboratory of Bioactive Substance and Function of Natural Medicines, Institute of Materia Medica, Chinese Academy of Medical Sciences & Peking Union Medical College, Beijing 100050, China; lvtingting@imm.ac.cn (T.L.); zhangzirui@imm.ac.cn (Z.Z.); yhy@imm.ac.cn (H.Y.); renshuyue@imm.ac.cn (S.R.); wangjingrong@imm.ac.cn (J.W.); lishang@imm.ac.cn (S.L.); 2 Beijing Key Laboratory of Drug Target Identification and New Drug Screening, Institute of Materia Medica, Chinese Academy of Medical Sciences & Peking Union Medical College, Beijing 100050, China

**Keywords:** pituitary adenomas, genomics, tamoxifen, apoptosis, migration, tumor-associated macrophages

## Abstract

Although pituitary adenomas are histologically benign, they are often accompanied by multiple complications, such as cardiovascular disease and metabolic dysfunction. In the present study, we repositioned the Food and Drug Administration -approved immune regulator tamoxifen to target STAT6 based on the genomics analysis of PAs. Tamoxifen inhibited the proliferation of GH3 and AtT-20 cells with respective IC_50_ values of 9.15 and 7.52 μM and increased their apoptotic rates in a dose-dependent manner. At the molecular level, tamoxifen downregulated phosphorylated PI3K, phosphorylated AKT and the anti-apoptotic protein Bcl-2 and increased the expression of pro-apoptotic proteins p53 and Bax in GH3 and AtT-20 cells. Furthermore, tamoxifen also inhibited the migration of both cell lines by reprogramming tumor-associated macrophages to the M1 phenotype through STAT6 inactivation and inhibition of the macrophage-specific immune checkpoint SHP1/SHP. Finally, administration of tamoxifen (20, 50, 100 mg·kg^−1^·d^−1^, for 21 days) inhibited the growth of pituitary adenomas xenografts in nude mice in a dose-dependent manner. Taken together, tamoxifen is likely to be a promising combination therapy for pituitary adenomas and should be investigated further.

## 1. Introduction

Pituitary adenoma (PA) is the second-most common intracranial neuroendocrine malignancy, accounting for about 15–25% of all intracranial neoplasms [1,2]. Although PAs are histologically benign [3], hypersecretion of pituitary hormones from the tumor masses can result in multiple complications by acting in a paracrine manner. Cardiovascular diseases are the primary complications associated with PAs, which can trigger metabolic dysfunction and impair quality of life. Furthermore, 34–60% of the PAs are invasive [3], which makes surgical resection challenging and leads to high recurrence rates [4]. The benefits of chemotherapy and radiotherapy are also minimal for PA patients [5,6,7]. The currently marketed drugs for PAs can only relieve some symptoms caused by abnormal hormone secretion, but cannot inhibit tumor growth, thereby warranting further drug development.

Tamoxifen (TAM) was approved by the Food and Drug Administration (FDA) for the treatment of breast cancer in 1977, and has been used in clinical trials for glioma, liver cancer and endometrial cancer [8]. Furthermore, in vitro studies have shown that, TAM promotes cell cycle arrest, apoptosis and antiangiogenic activity of cancer cell lines, and reduces proliferation rates [9]. Although TAM inhibits the growth of PAs as an estrogen antagonist [10], the effect of TAM on the plasticity of tumor-associated macrophages, and its bearing on the inhibition of PAs are still unclear.

The phosphatidylinositol-3-kinase (PI3K)/protein kinase B (AKT) signaling pathway is associated with the proliferation, growth, and invasion of PAs [11,12,13]. In addition, M2-like polarized tumor-associated macrophages also play key roles in promoting tumors cell growth, invasion, metastasis and angiogenesis [14]. Thus, the M2 macrophages are a promising target for cancer therapy. Gefitinib (GEFI) significantly inhibits the M2-like polarization of tumor-associated macrophages in Lewis lung cancer xenografts, thereby inhibiting tumor cell invasion and migration [15,16]. 

In this study, we repositioned TAM to target signal transducer and activator of transcription 6 (STAT6) based on the analysis of the gene expression profiles of PAs. TAM induced apoptosis of the PAs cells and changed the expression levels of apoptosis-related proteins in these cells. In addition, TAM also inhibited the migration of the GH3 and AtT-20 cell lines by reprogramming macrophages to the M1 phenotype via STAT6 inactivation and blockade of the macrophage-specific immune checkpoint src-homology domain 2 (SH2)-containing protein tyrosine phosphatase (SHP)-SHP1/SHP2. 

## 2. Results

### 2.1. Pituitary Adenomas Progression Correlates with STAT6 Expression Levels

**Figure 1:**
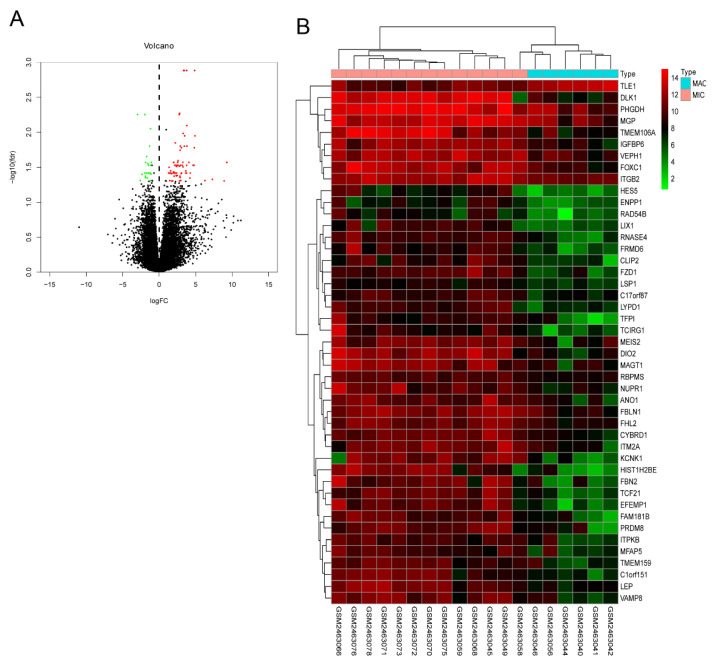
Volcano and heat maps of DEGs between MACs and MICs. (**A**) Volcano map of DEGs between MACs and MICs (|log FC| ≥ 1 and corrected *p*-value < 0.05). (**B**) Hierarchical clustering heatmap of DEGs between MACs and MICs screened on the basis of log FC > 1 and corrected *p*-value < 0.05 (upregulated genes are in red; downregulated genes are in blue; nonregulated genes are in black). DEGs: differentially expressed genes; MACs: macroadenomas; MICs: microadenomas.

**Figure 2:**
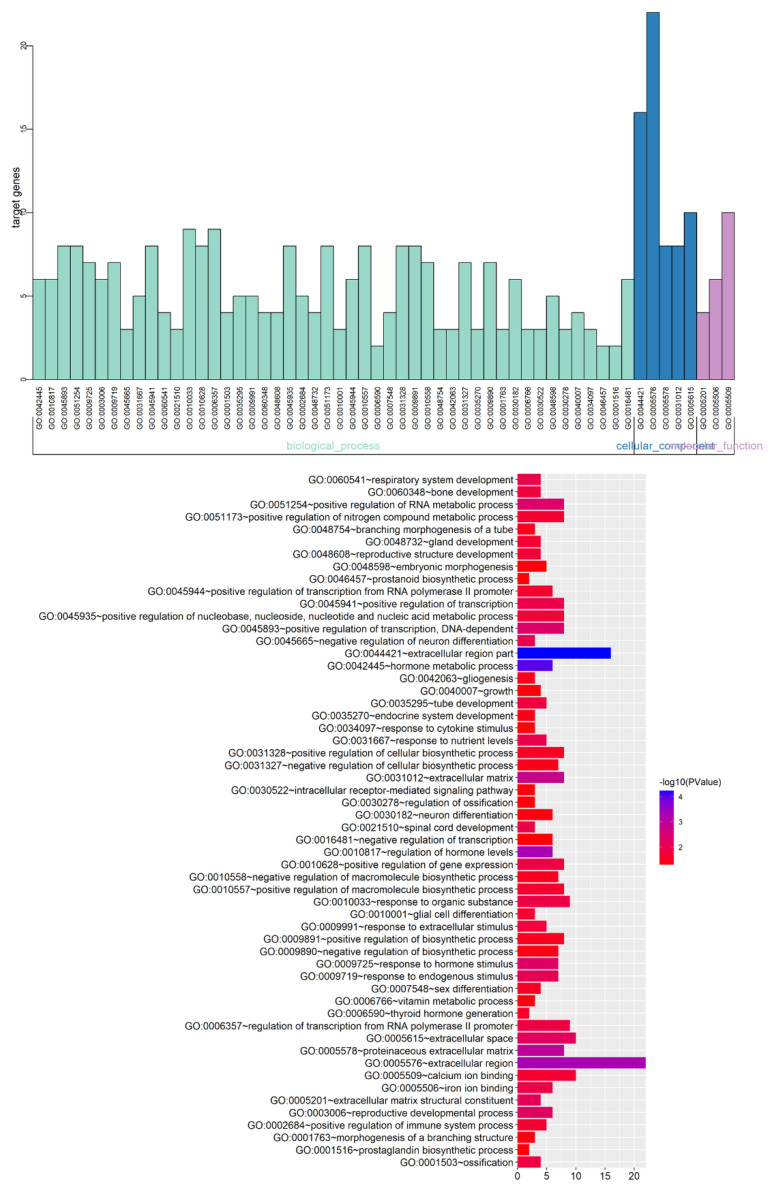
GO and pathway enrichment analysis of DEGs between MACs and MICs. DEGs were functionally annotated into molecular function, biological processes and cell composition groups. GO: gene ontology; DEGs: differentially expressed genes; MACs: macroadenomas; MICs: microadenomas.

**Figure 3:**
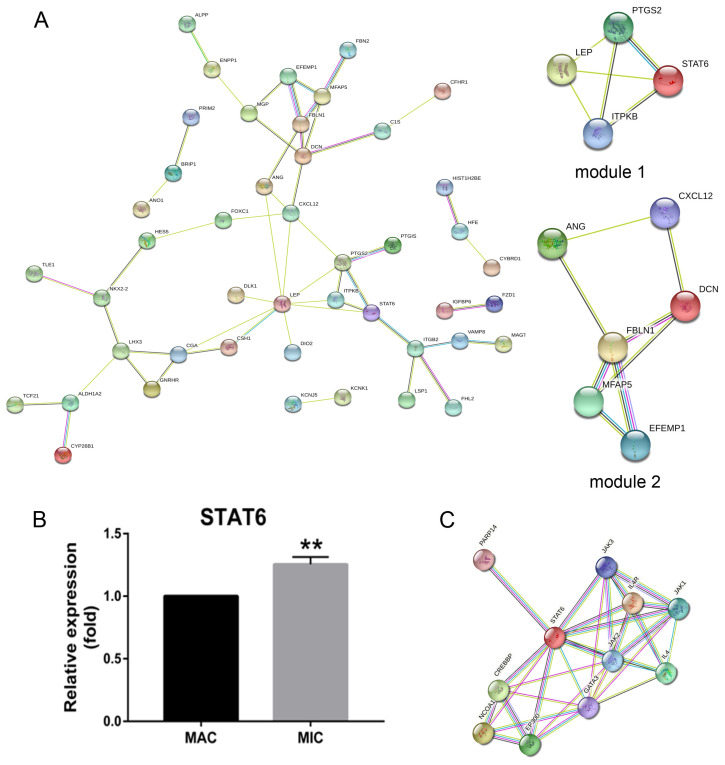
Activated STAT6 is positively associated with the progression of PAs. (**A**) PPI network and top two modules of DEGs. (**B**) STAT6 expression levels in MACs and MICs tissues quantified by qRT-PCR. Bars indicates SEM, *p*-values represent the significant difference between MACs and MICs. (**C**) Direct interaction module obtained from the STAT6-centred PPI network. Line thickness and colors represent network, and node size and colors represent the degree. ** *p* < 0.01. PAs: pituitary adenomas; DEGs: differentially expressed genes; MACs: macroadenomas; MICs: microadenomas; PPI: protein-protein interaction.

To explore the genetic basis of PA growth and progression, we analyzed the gene expression profiles of macroadenomas (MACs) and microadenomas (MICs) in the GSE93825 dataset from the Gene Expression Omnibus (GEO) database. There were 96 differentially expressed genes (DEGs) between MACs and MICs, including 25 upregulated and 71 downregulated genes (Figure 1A) with statistical significance (corrected *p*-value < 0.05, log FC > 1). The top 45 DEGs (Figure 1B) were functionally annotated using the Database for Annotation, Visualization and Integrated Discovery (DAVID; http://david.ncifcrf.gov, accessed on 13 November 2020) (Table 1) and Enrich chip annotation tools (Figure 2). A protein–protein interaction (PPI) network was then constructed (Figure 3A), and the top five hub genes included *leptin (LEP), prostaglandin-endoperoxide synthase 2 (PTGS2), STAT6, C-X-C motif chemokine ligand 12 (CXCL12)* and *inositol-trisphosphate 3-kinase B (ITPKB)* (Table 2). The mRNA expression levels of these hub genes were validated through qRT-PCR, which showed a positive correlation between the expression levels of STAT6 and the progression of PAs (Figure 3B), indicating STAT6 is a potential therapeutic target. The STAT6-centered network is outlined in Figure 3C.

### 2.2. Repositioning of Tamoxifen to Target STAT6 to Control the Progression of Pituitary Adenomas

**Figure 4:**
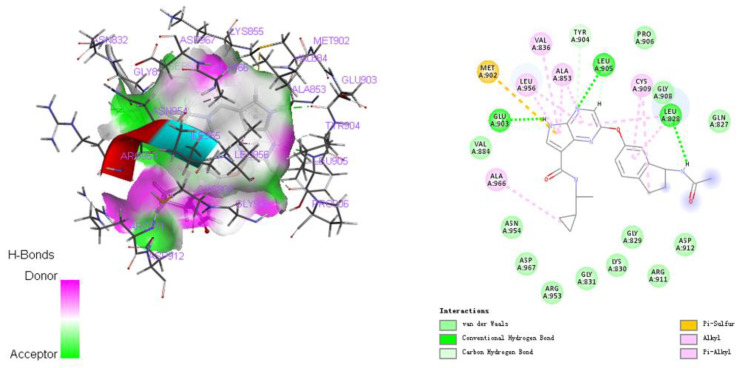
The docking mode between TAM and STATTAM: tamoxifen.

Based on the results above, we repositioned TAM to target STAT6 in order to develop a new potential drug for PAs. “STAT6” was input as a target in the Drugbank database, and TAM was identified as a STAT6-targeting drug. The molecular docking between TAM and STAT6 was confirmed using the CHARMM-based CDOCKER program. The docking mode between TAM and STAT6 is shown in Figure 4, and the docking energy is summarized in Table 3.

### 2.3. Tamoxifen Inhibited Pituitary Adenomas Progression Both In Vitro and In Vivo

**Figure 5:**
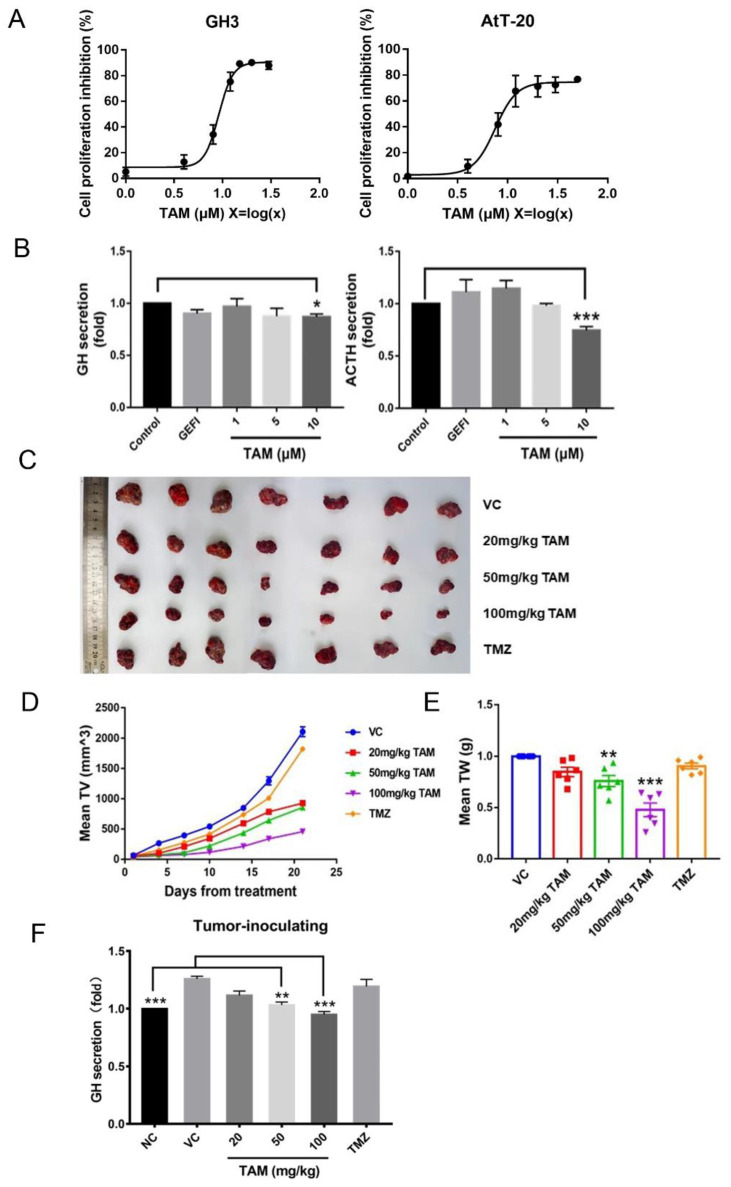
TAM inhibited PAs progression both in vitro and in vivo. (**A**) IC_50_ values were determined by CCK8 assay after GH3 and AtT-20 cells treatment with TAM. (**B**) The treatment of TAM reduced the levels of GH and ACTH secreted by the GH3 and AtT-20 cells respectively. (**C**) Xenograft mouse model establishment and TAM administration. The panel showed representative tumor tissues of animals at the end of treatment. (**D**) TAM inhibited tumor growth as measured by tumor volume. (**E**) TAM inhibited tumor growth as measured by tumor weight. (**F**) The secretion levels of GH in the plasma were detected using an enzyme-linked immunosorbent assay. NC normal control, sham mice treated with the vehicle. VC vehicle control, refers to tumor-bearing mice treated with the vehicle. Bars indicates SEM, * *p* < 0.05, ** *p* < 0.01, *** *p* < 0.001 vs. control, VC or NC. TAM: tamoxifen; PAs: pituitary adenomas; GEFI: gefitinib; TMZ: temozolomide.

As shown in Figure 5A, TAM inhibited the proliferation of GH3 and AtT-20 cells after 24 h of culture, and the IC_50_ doses were 9.15 μM and 7.52 μM respectively. In addition, TAM treatment significantly reduced the levels of growth hormone (GH) and adrenocorticotropic hormone (ACTH) secreted by the GH3 and AtT-20 cells respectively (Figure 5B), while GEFI treatment did not significantly affect hormone secretion. 

GH3 cells were inoculated subcutaneously into BALB/c-nu nude mice to establish adenoma xenografts, and the mice were treated with different doses of TAM or temozolomide (TMZ) for 21 days (Figure 5C). TAM treatment not only led to a significant reduction in tumor volume and weight (Figure 5D,E), but also decreased the plasma levels of GH in the tumor-bearing mice (Figure 5F). The inhibitory effect of TAM was dose-dependent and stronger than that of TMZ.

### 2.4. Tamoxifen Induced Apoptosis of the Pituitary Adenomas and Changed the Expression Levels of Apoptosis-Related Proteins in Pituitary Adenomas

**Figure 6:**
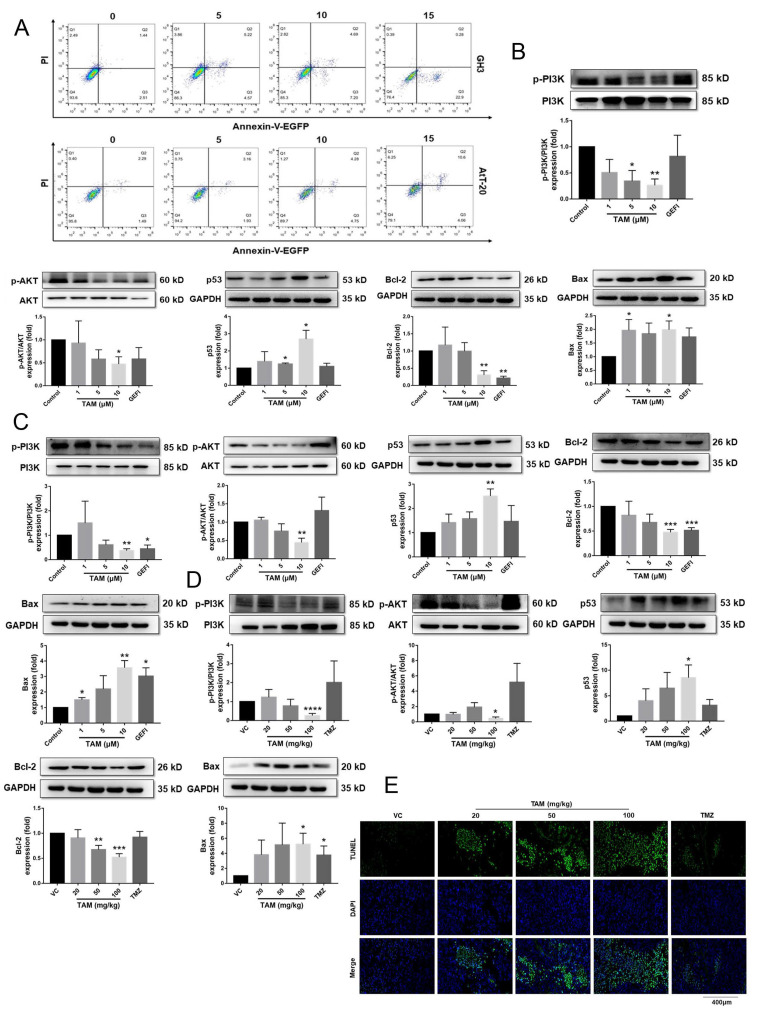
TAM induced apoptosis of PAs and changed the expression levels of apoptosis-related proteins in PAs. (**A**) Flow cytometric analysis of apoptosis with cells double stained with Annexin V-EGFP/PI, showing that TAM increased the apoptosis rates of GH3 and AtT-20 cells. (**B**,**C**) The expression levels of p53, Bcl-2 and Bax, the phosphorylation levels of PI3K and AKT normalized to GAPDH in GH3 and AtT-20 cells were detected using Western blot. (**D**) The key protein expression levels of the PI3K/AKT signaling normalized to the GAPDH in tumor tissues were detected using Western blot. (**E**) Representative tumor tissue sections with TUNEL staining of apoptotic cells (green). Cell nuclei were stained with DAPI (blue). Images are at 40× magnification. VC vehicle control, refers to tumor-bearing mice treated with the vehicle. Bars indicates SEM, * *p* < 0.05, ** *p* < 0.01, *** *p* < 0.001, **** *p* < 0.0001 vs. control or VC. TAM: tamoxifen; PAs: pituitary adenomas; GEFI: gefitinib; TMZ: temozolomide.

The apoptosis rates of the cultured cells were analyzed by Annexin V-EGFP/PI double staining. As shown in Figure 6A, TAM induced apoptosis of GH3 and AtT-20 cells in a dose-dependent manner. Furthermore, TAM treatment also downregulated p-PI3K, p-AKT and the anti-apoptotic B-cell lymphoma-2 (Bcl-2) protein, and upregulated the pro-apoptotic p53 and Bcl-2 associated X protein (Bax) in both cells lines compared to the untreated controls, whereas GEFI had no significant effect on the expression levels of apoptosis-related proteins (Figure 6B,C and Figure S1A).

Similar changes were observed in the in situ expression of the PI3K/AKT pathway proteins in xenograft tissues of different groups (Figure 6D and Figure S1B). Furthermore, a TUNEL assay revealed a marked increase in the number of apoptotic cells in the TAM-treated versus untreated tumors (Figure 6E and Figure S2), whereas TMZ had no significant effect on the apoptosis of tumor cells.

Taken together, TAM may inhibit progression of PAs by inducing apoptosis and expression changes of apoptosis-related proteins.

### 2.5. Tamoxifen Inhibited the Migration of Pituitary Adenomas Cells by Inducing M1 Polarization of Tumor-Associated Macrophages via STAT6 Inactivation and SHP1/SHP2 Blockade

**Figure 7:**
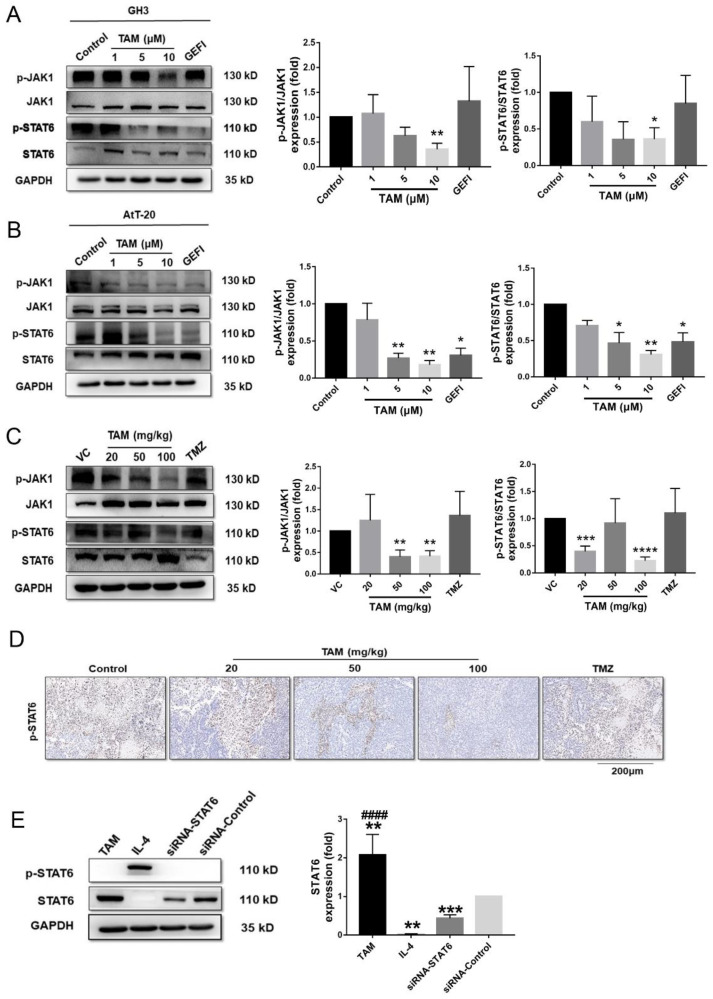
TAM inhibited activation of immune-related factor STAT6. (**A**,**B**) The phosphorylation levels of JAK1 and STAT6 normalized to GAPDH in GH3 and AtT-20 cells were detected using Western blot. (**C**) The phosphorylation levels of JAK1 and STAT6 normalized to GAPDH in tumor tissues were also detected. (**D**) Immunohistochemical (IHC) staining of p-STAT6 in representative tumor tissues sections. Images are at 20× magnification. (**E**) The p-STAT6 and STAT6 protein expression levels normalized to the GAPDH in RAW 264.7 cells were detected. Bars indicates SEM, VC vehicle control, refers to tumor-bearing mice treated with the vehicle. * *p* < 0.05, ** *p* < 0.01, *** *p* < 0.001, **** *p* < 0.0001, #### *p* < 0.0001 vs. control or VC. TAM: tamoxifen; GEFI: gefitinib; TMZ: temozolomide.

To further delineate the cellular and molecular mechanisms underlying the inhibitory action of TAM against PAs, we analyzed its effect on STAT6 expression levels. TAM downregulated p-Janus kinase 1 (JAK1) and p-STAT6 in the GH3 and AtT-20 cells in a dose-dependent manner, whereas no significant changes were observed in the GEFI-treated cells (Figure 7A,B and Figure S3A). Likewise, the medium and high doses of TAM significantly decreased the expression of p-JAK1 and p-STAT6 in the tumor tissues compared to that of the untreated control group, and the effect was stronger than that of TMZ (Figure 7C,D), as detected by Western blotting (Figure S3B) and immune histology chemistry (IHC) (Figure S4). Additionally, Figure 7E showed that compared with siRNA-control, the expression of p-STAT6 was also significantly decreased in RAW 264.7 cells treatment with TAM, whereas dramatically increased in cells treatment with IL-4 (Figure S5A).

**Figure 8:**
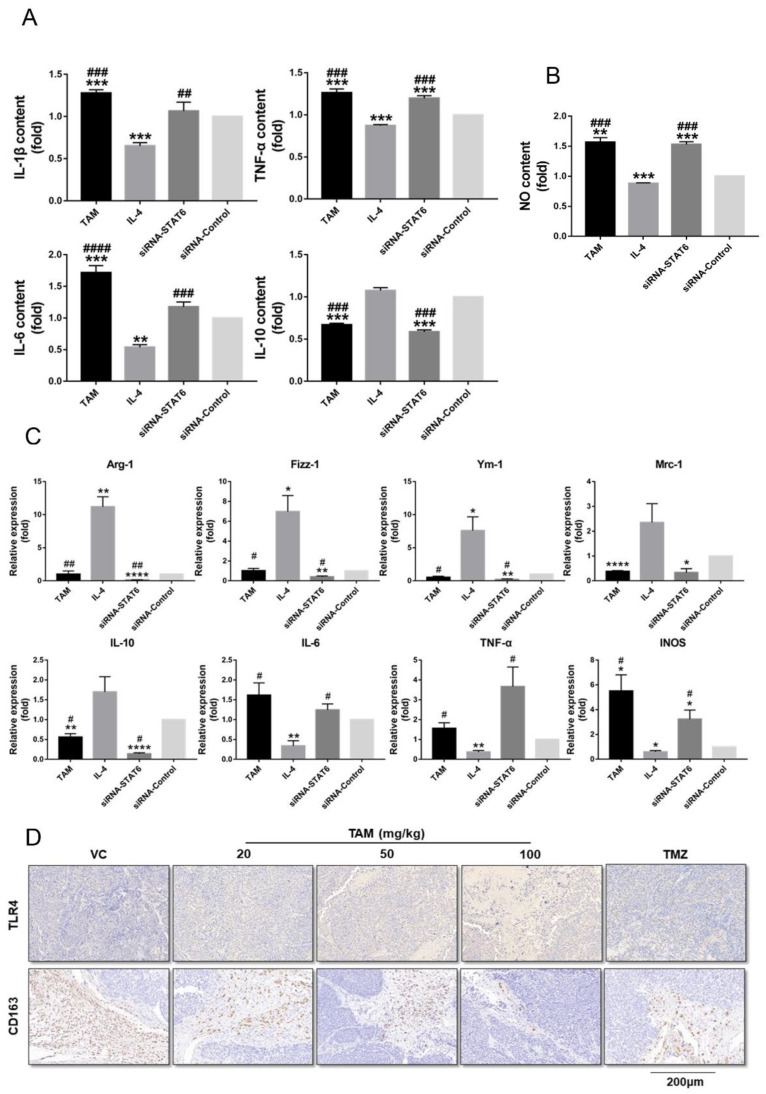
TAM inhibited polarization of M2 macrophages induced by STAT6. (**A**) The expression levels of IL-1β, IL-6, TNF-α and IL-10 in the supernatant of RAW 264.7 cells were detected using an ELISA kit according to the manual guide. (**B**) The content of nitric oxide released in the supernatant of RAW 264.7 cells treated with TAM or IL-4. (**C**) TAM inhibited the expression of specific M2-marker genes and increased the expression of specific M1-marker genes. qRT-PCR was performed to assess the mRNA levels of M1/2-marker genes. (**D**) IHC staining of TLR4+ and CD163+ macrophages in representative tumor tissue sections. Images are at 20× magnification. Bars indicates SEM, VC vehicle control, refers to tumor-bearing mice treated with the vehicle. * *p* < 0.05, ** *p* < 0.01, *** *p* < 0.001, **** *p* < 0.0001 vs. control or VC. # *p* < 0.05, ## *p* < 0.01, ### *p* < 0.001, #### *p* < 0.0001 vs. cells treated with IL-TAM: tamoxifen; IHC: immune histology chemistry.

Since IL-4 mediates M2 polarization through STAT6 phosphorylation, we next determined whether TAM also affects the phenotypic polarization of macrophages. As shown in Figure 8, TAM exposure inhibited M2 polarization of the RAW 264.7 cells to a similar extent as STAT6 silencing. Compared to the untreated control, the TAM-treated cells secreted significantly higher levels of IL-1β, IL-6 and TNF-α after 24 h, whereas IL-4 stimulation increased IL-10 secretion (Figure 8A). In addition, the amount of nitric oxide released in the supernatant was markedly higher for the TAM-treated or STAT6-silenced macrophages compared to the IL-4 group (Figure 8B). To further confirm the impact of TAM on M2 polarization, we analyzed the transcriptional changes in specific M1 or M2 marker genes. Compared to the IL-4-treated group, TAM treatment or STAT6 silencing significantly downregulated M2 genes such as *mannose receptor C type-1 (Mrc-1)*, *chil3 (Ym-1)*, *Retnla (Fizz-1)*, *arginase-1 (Arg-1)* and *IL-10*, and upregulated the M1 genes including *IL-6*, *TNF-α* and *inducible nitric oxide synthase* (*INOS*) in the macrophages (Figure 8C). Consistent with this, TAM treatment markedly increased the number of Toll-like receptors 4 (TLR4+) macrophages and decreased that of CD163+ macrophages in the tumor tissues (Figure 8D). These results suggest that TAM may inhibit M2 polarization by inactivating STAT6.

**Figure 9:**
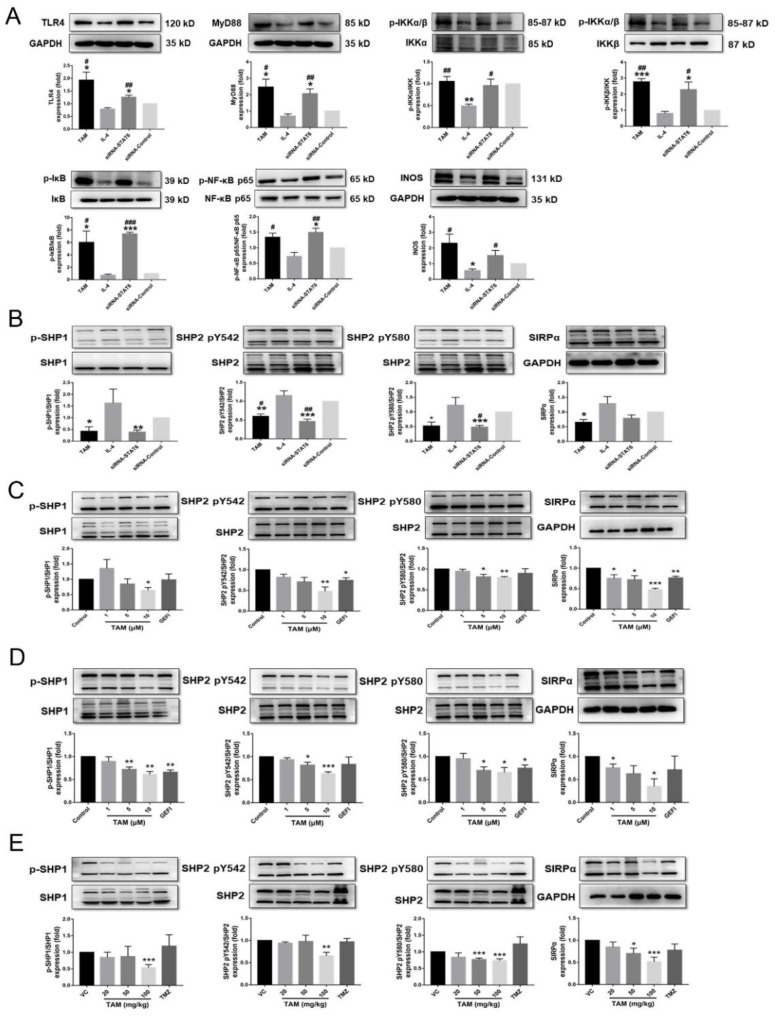
TAM activated TLR4 signaling and inactivated immune checkpoint SHP1/SHP2. (**A**) The protein expression levels of the TLR4/NF-κB signaling pathway normalized to the GAPDH in RAW 264.7 cells were detected. (**B**) The expression levels of SIRPα protein, the phosphorylation levels of SHP1 and SHP2 proteins in RAW 264.7 cells were detected using Western blot. (**C**,**D**) The expression levels of SIRPα protein, the phosphorylation levels of SHP1 and SHP2 proteins in GH3 and AtT-20 cells were detected. (**E**) The expression levels of SIRPα protein, the activity of SHP1 and SHP2 proteins in tumor tissues were also detected. Bars indicates SEM, VC vehicle control, refers to tumor-bearing mice treated with the vehicle. * *p* < 0.05, ** *p* < 0.01, *** *p* < 0.001 vs. control or VC. # *p* < 0.05, ## *p* < 0.01, ### *p* < 0.001 vs. cells treated with IL-TAM: tamoxifen.

Furthermore, the expression of the TLR4 protein was significantly upregulated upon treatment with STAT6 inhibitor-TAM compared to the STAT6-induced M2 macrophages (Figure 9A and Figure S5A). TAM-mediated upregulation of the TLR4 protein in the M1 macrophages was associated with increased expression levels of myeloid differentiation factor 88 (MyD88) and INOS protein, along with increased phosphorylation of IκB kinase (IKK), inhibitor of NF-κB (IκB) and nuclear factor κB (NF-κB) p65 protein (Figure 9A and Figure S5A). In addition, macrophages treated with TAM expressed lower levels of phosphorylated immune checkpoints SHP1 and SHP2, as well as signal regulatory proteins α (SIRPα) (Figure 9B and Figure S5B). SHP1/SHP2 activity and the expression level of SIRPα were also significantly reduced in TAM-treated GH3 and AtT-20 cells as well as tumor tissues of mice (Figure 9C–E and Figure S5C–E). However, TMZ had no significant effect on macrophage polarization or immune checkpoint activity.

**Figure 10:**
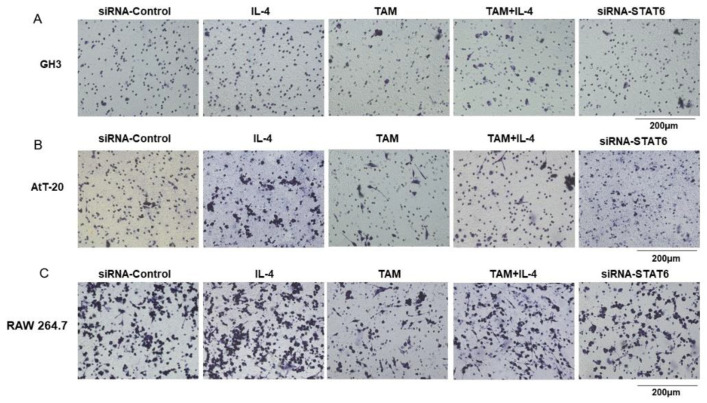
TAM abrogated the M2 macrophage effects on the migration of GH3 and AtT-20 cells. (**A**–**C**) The GH3, AtT-20 as well as RAW 264.7 cells were seeded in the upper chamber and RAW 264.7 cells treated with IL-4, TAM or siRNA-STAT6 were seeded in the lower chambers and allowed to incubate at 37 °C with 5% COThe migrated cells on the bottom chamber were stained with hematoxylin. Then, the effect of different polarization states of macrophages on RAW 264.7, GH3 and AtT-20 cells migration was evaluated by Transwell assay. Images are at 20× magnification. TAM: tamoxifen.

To assess the effect of TAM on the interaction between tumor cells and functional macrophages, the GH3, AtT-20 or RAW 264.7 cells were respectively co-cultured with RAW 264.7 cells, and their migration abilities under different polarization states of macrophages were determined by the Transwell assay. Compared to the untreated controls, IL-4-treated M2 macrophages significantly enhanced the migration of GH3, AtT-20 and RAW 264.7 cells. In contrast, macrophages treated with TAM or TAM combined with IL-4 had an inhibitory effect on migration (Figure 10A–C), suggesting that TAM inhibited tumor cell migration by abrogating M2 polarization of tumor-associated macrophages and immune checkpoint SHP1/SHP2 blockade. 

## 3. Discussion

Through bioinformatics analysis, we found that the activity and expression level of STAT6 correlated with the size of PAs. In addition, STAT6 was predicted as one of the targets of TAM, which led us to reposition the drug to target STATTAM inhibited the proliferation and migration of two PA cell lines in vitro and in vivo, and induced apoptosis and expression changes of apoptosis-related proteins. In addition, the inhibitory effect of TAM on tumor cell migration was associated with the blockade of the macrophage-specific immune checkpoint SHP1/SHP2, and reprogramming of the tumor-associated macrophages to the pro-inflammatory M1 phenotype via STAT6-inactivation.

The correlation between aberrant STAT6 activity/expression levels and the size of the PAs suggested that overactivation of STAT6 increased the risk of developing MACs as opposed to MICs. Previous studies have shown that STAT6 is associated with the tumorigenesis, immunosuppression, proliferation, metastasis and poor prognosis of human cancers [17]. For example, STAT6 activation promotes the malignant behavior of colon cancer, prostate cancer, breast cancer and mediastinal large B-cell lymphoma cells [15]. Several miRNAs have been identified that act as tumor suppressors in prostate cancer by targeting STAT6, and inhibit the growth and metastasis of tumor cells, and induce apoptosis [18,19,20]. Furthermore, genome-wide DNA methylation and mRNA microarray analysis of 68 PA patients indicated that STAT6 expression and methylation may be associated with the invasiveness of PA cells [21]. In our study, we found that STAT6 activity was significantly inhibited in the PA cells and tissues after TAM treatment, suggesting that STAT6 may be a potential diagnostic marker and therapeutic target.

Apoptosis is an innate tumor suppression mechanism that inhibits tumorigenesis at multiple stages, including transformation and metastasis [22]. TAM induces apoptosis in breast cancer cells by triggering cytochrome C release and activating caspase family proteins [10]. Another study showed that TAM promotes apoptosis of C6 glioma cells by silencing the PI3K/AKT signaling pathway [23]. In addition, the PI3K/AKT pathway is constitutively activated in many tumors, including PAs, and plays a key role in tumorigenesis and progression [24,25,26]. In the present study as well, TAM induced apoptosis of PA cells and changed the expression levels of apoptosis-related proteins in these cells.

TAM is also known to regulate phenotypic polarization of macrophages in PAs. Tumor-associated macrophages stimulate angiogenesis, tumor progression and metastasis by suppressing immune responses [7,27], and are polarized to the anti-inflammatory M2 phenotype [28]. The M2 macrophages are abundant in hepatocellular carcinoma tumors, and correlate with the activation of the JAK1/STAT6 signaling pathway [29]. M2 macrophages also promote triple-negative breast cancer progression through the JAK1/STAT1 pathway [30]. We found that the ratio of M1/M2 macrophages increased significantly in the tumor tissues after TAM treatment, as indicated by the downregulation in M2 markers. Likewise, TAM exposure induced the pro-inflammatory M1 phenotype similar to the LPS-mediated reprogramming of M2 macrophages to the M1 phenotype [31,32]. STAT6 activation on the other hand had an opposite effect. Furthermore, TAM treatment also reprogrammed the IL-4-stimulated M2 macrophages and led to a decrease in STAT6 activity, and STAT6-knockdown macrophages were more inclined to polarize towards the M1 phenotype. Taken together, TAM inhibits M2 polarization of macrophages by inactivating STAT6 signaling.

Macrophages are highly plastic and exhibit distinct functional phenotypes in response to cytokines, pathogens and other stimuli [33,34]. M1 macrophages are pro-inflammatory, immunogenic and anti-oncogenic, whereas the M2 macrophages are anti-inflammatory, tolerogenic and oncogenic [22,33,35]. M2 macrophages induce migration and invasion of colon cancer and hepatocellular carcinoma cells [36,37]. In addition, conditional media collected from M2-like polarized macrophages promoted the invasion and proliferation of primary cells from non-functioning PAs [38]. Consistent with these previous reports, we found that TAM-treated macrophages significantly lesser the extent of GH3 and AtT-20 cell migration compared to the controls. Thus, TAM may inhibit PA growth and progression by modulating the tumor microenvironment. 

The therapeutic efficacy of several classical anticancer drugs is dependent on their ability to modify the immune response in the tumor milieu [10,39,40]. The immunostimulatory properties of TAM have been established previously. For example, TAM suppressed brain metastasis of estrogen receptor-deficient breast cancer cells by blocking polarization of M2 microglia and enhancing an anti-tumor immune response [41]. Furthermore, TAM reduced fibrosis in pancreatic ductal adenocarcinoma tissues and regulated inflammatory and immune responses by directing macrophage polarization [33].

Immune checkpoint blockers are increasingly being considered for cancer treatment. For instance, phomoxanthone A and B reduced the proliferation of MCF7 cells by inhibiting SHP1 activity [42]. In another study, compound 25 blocked the motility and growth of various cancer cells via selective inhibition of SHP2 [10]. Moreover, one patient with corticotroph carcinoma showed a significant reduction in hormone levels and tumor shrinkage immediately after treatment with the PD-1/PD-L1 blockers ipilimumab and nivolumab [43], while another patient progressed rapidly after four cycles of pembrolizumab (PD-1 blocker) treatment [27]. In the present study, we found that TAM can blocked the macrophage-specific immune checkpoint SHP1/SHP2 to inhibit the migration and progression of PAs. There are currently no anti-PA drugs that specifically target SHP1/SHP2, which raises the possibility of using TAM to boost the clinical outcomes for PA patients.

The limitations in the present study should also be noted here. First, the PK/PD data of TAM in pituitary adenoma/carcinoma patients have not been reported so far, however we noted that TAM can penetrate the blood-brain barrier (BBB). For example, Lien et al. determined that the concentration of TAM and its metabolites in the brain tissue of patients with brain metastases from breast cancer was 46-fold higher than serum levels [44]. Apart from that, the only known PKC inhibitor that can penetrate the BBB is the selective estrogen receptor modulator TAM, which can significantly reduce manic symptoms in manic patients in a short period of time (3–7 days) [45]. In addition, although only four cases of TAM activity against PAs have been reported in trials over decades, these PA patients experienced reductions in tumors size and normalization of hormone levels after taking TAM [46,47,48,49]. Furthermore, our current study of the treatment and mechanism of action of GH- and ACTH-type PAs has limited understanding of the differential importance of these pathways in various subtypes of PAs. Finally, in this study, we propose for the first time that TAM may inhibit the migration of PA cells by regulating macrophage phenotype polarization and macrophage-specific immune checkpoints, but it is still necessary to further study the specific mechanism of the effect of M1/M2 macrophages on PA cells and the verification of the inhibition of SIRPα-CD47 binding, and determine the rational drug combination for future drug development.

## 4. Materials and Methods

### 4.1. Gene Expression Profiles Analyses and Validation of the Hub Genes

The GSE93825 microarray dataset was downloaded from GEO database (Table 4). The DEGs between MACs and MICs were screened using GEO2R, and volcano maps and heat maps were plotted using “R” software (version 4.0.0) (University of Auckland, Auckland, New Zealand). DAVID (version 6.8) was used to perform GO and pathway enrichment analyses, and the PPI network was conducted with the Search Tool for the Retrieval of Interacting Genes (STRING) (version 11.0) and visualized using Cytoscape (Institute of Systems Biology, University of California, San Diego, CA, USA). Important modules and hub genes in the PPI network were identified using the molecular complex detection (MCODE) plugin. The hub genes were verified by quantifying the mRNA expression levels through qRT-PCR. 

### 4.2. Drug Repositioning and MOLECULAR Docking

The drugs directed to specific targets were retrieved from the DrugBank database (https://www.drugbank.ca/, accessed on 1 March 2021). Next, the FDA-approved targeted STAT6 drug, TAM, was confirmed by docking analysis via CDOCKER. Specifically, molecular docking was performed using Discovery Studio 2018 software (BIOVIA, San Diego, CA, USA) based on the protein data bank (PDB) and Pubchem database. The crystal structure of STAT6 was retrieved from PDB (ID: 3ZEP); wherein the resolution of protein structure was 2.35 Å. Preparation of compound and proteins: TAM was prepared with prepare ligands module of small molecules. Then we performed a preparation of STAT6 protein following the same protocol as in our previous studies [50]. The prepared protein was defined as the receptor and the binding sites were defined from PDB site records with the define and edit binding site module. Finally, TAM was docked into the binding site spheres of 3ZEP by utilizing the CDOCKER modules.

### 4.3. Cell Culture and Proliferation Assay

The murine pituitary corticotrophin tumor AtT-20 cell line (ATCC CRL-1795) was purchased from the American Type Culture Collection (ATCC, Manassas, VA, USA). Rat pituitary tumor GH3 cells and murine RAW 264.7 macrophages were preserved in our laboratory. The AtT-20 and RAW 264.7 cells were cultured in Dulbecco’s modified Eagle’s medium (DMEM) (Gibco BRL, Grand Island, NY, USA) supplemented with 10% fetal bovine serum (FBS) (Gibco BRL, Grand Island, NY, USA), 100 IU/mL penicillin and 100 μg/mL streptomycin (Solarbio, Beijing, China), and GH3 cells were maintained in Ham’s F-12K medium (Procell, Wuhan, Hubei, China) containing 15% horse serum (Gibco BRL, Grand Island, NY, USA), 2.5% FBS, 100 IU/mL penicillin and 100 μg/mL streptomycin. All cells were cultured at 37 °C in a humidified incubator under 5% CO_2_.

GH3 and AtT-20 cells were seeded in 96-well plates at a density of 2 × 10^4^ cells per well and incubated with the drugs at appropriate concentrations for 24 h. The percentage of viable cells was determined using the Cell Counting Kit-8 (KeyGen Biotech, Nanjing, Jiangsu, China) according to the manufacturer’s instructions, and measuring the absorbance at 450 nm. IC_50_ values were calculated using GraphPad Prism 7 software (GraphPad Software Inc, San Diego, CA, USA).

### 4.4. Measurement of Hormone Levels

GH3 and AtT-20 cells were seeded in the 60 mm dishes at the density of 5 × 10^5^ cells/dish and cultured with different concentrations of TAM (MCE, Shanghai, China) or 10 μM GEFI (MCE, Shanghai, China) for 24 h. GH (CSB-E07343m) and ACTH (CSB-E06874m) levels in the supernatant of cultured cells and plasma of tumor-bearing mice were detected by enzyme-linked immuno-sorbent assay (ELISA) using specific kits (CUSABIO Biotech Co., Ltd, Wuhan, Hubei, China) according to the manufacturer’s instructions.

GEFI was used as the positive control since it is a selective inhibitor of epidermal growth factor receptor (EGFR) and inhibits the growth of PA cells by blocking EGFR-mediated ACTH secretion [51,52].

### 4.5. Annexin V-EGFP/PI Staining

Apoptotic cells were detected using the Annexin V-EGFP cell apoptosis detection kit (KeyGen Biotech, Nanjing, Jiangsu, China) according to the manufacturer’s instructions. Briefly, GH3 and AtT-20 cells were cultured in 6-well plates with 0, 5, 10, and 15 μM TAM for 24 h and harvested. The cells were then incubated with Annexin V-EGFP and PI for 30 min at room temperature, and analyzed by flow cytometry. The percentage of apoptotic cells was calculated using Flow Jo software (Tristar, CA, USA).

### 4.6. SiRNA Transfection

Small interfering RNA (siRNA) specific for STAT6 (siRNA-STAT6) and a scrambled control were obtained from Genepharma (Shanghai, China) and transfected into RAW 264.7 cells using Lipofectamine 3000 (Thremo, Waltham, MA, USA) according to the manufacturer’s instructions. The sequence of siRNA-STAT6 was 5′-GGTTCAGATGCTTTCTGTT-3′. 

### 4.7. Detection of Macrophage Polarization

RAW 264.7 cells were treated with 20 ng/mL IL-4 (067258) (Cell Signaling, Danvers, MA, USA) or 10µM TAM. The expression levels of Mrc-1, Ym-1, Fizz-1, Arg-1, IL-10 (M2 markers), IL-6, TNF-α and INOS (M1 markers) mRNAs were detected by qRT-PCR. The levels of secreted IL-1β (CSB-E08054m), IL-6 (CSB-E04639m), TNF-α (CSB-E04741m) and IL-10 (CSB-E04594m) were measured using specific ELISA kits (CUSABIO Biotech Co., Ltd, Wuhan, Hubei, China). The amount of nitric oxide released into the supernatant was measured using a colorimetric assay kit (Applygen Technologies Inc., Beijing, China).

### 4.8. Transwell Assay

A Transwell Boyden chamber with polycarbonate filters of diameter 6.5 mm and pore size 8 μm (Costar, Bethesda, MD, USA) was used to perform the migration assay. RAW 264.7 cells were seeded in the lower chambers at a density of 2 × 10^5^ per well in complete DMEM for 24h, the medium was replaced with fresh DMEM supplemented with 1% FBS and IL-4 with or without TAM. Then, after 24 h of culture, we replaced the medium with fresh DMEM supplemented with 1% FBS and only after then the RAW 264.7, GH3 and AtT-20 cells were respectively seeded in the upper compartment at the density of 2 × 10^5^ per well. After culturing for 24 h, the resident cells were removed with a moist cotton swab, and the cells that migrated to the lower compartment were fixed with 4% paraformaldehyde fix solution (Gene-protein link, Beijing, China) for 30 min and stained with hematoxylin (Beyotime, Shanghai, China). The stained cells were photographed with a high-content imaging system and counted in three random fields per well at 20× magnification.

### 4.9. Western Blotting

The suitable treated cells and tumor tissues were harvested and homogenized in the radio immunoprecipitation assay (RIPA) lysis buffer (Solarbio Biotech Co., Ltd. Beijing, China) supplemented with protease and phosphatase inhibitor cocktails (Thremo, Waltham, MA, USA). Western blotting was performed as per standard protocols, and the membranes were probed with antibodies targeting p-JAK1 (74129, Rt, 1:500), JAK1 (29261, Rt, 1:1000), p-STAT6 (56554, Rt, 1:500), p-PI3K (17366, Rt, 1:500), PI3K (4249, Rt, 1:1000), p-AKT (4060, Rt, 1:500), AKT (4685, Rt, 1:1000), p53 (48818, Ms, 1:1000), Bax (14796, Rt, 1:1000), p-NF-κB p65 (3033, Rt, 1:500), NF-κB p65 (8242, Rt, 1:1000), p-IκBα (2859, Rt, 1:500), IκBα (4814, Ms, 1:1000), p-IKKα/β (2697, Rt, 1:500), IKKα (11930, Ms, 1:1000), IKKβ (8943, Rt, 1:1000), p-SHP1 (8849, Rt, 1:500), SHP1 (26516, Rt, 1:1000), p-SHP2 (5431, Rt, 1:500), SHP2 (3397, Rt, 1:1000), SIRPα (47027, Rt, 1:1000) (Cell Signaling, Danvers, MA, USA), STAT6 (YT4454, Rt, 1:1000), MyD88 (YM33092, Ms, 1:1000) (Immunway, Plano, MA, USA), GAPDH (10494-1-AP, Ms, 1:1000), INOS (22226-1-AP, Rt, 1:1000) (Proteintech, Wuhan, Hubei, China), Bcl-2 (ab32124, Rt, 1:1000) (Abcam, Cambridge, MA, USA) and TLR4 (sc-293072, Ms, 1:1000) (santa cruz biotechnology, Silicon Valley, CA, USA)positive bands were detected using an ECL Kit (CWBIO, Beijing, China) and the Tanon Chemiluminescence Image Analysis System (Shanghai, China).

### 4.10. RNA Isolation and qRT-PCR

The total RNA of RAW 264.7 cells was isolated using Trizol (Thermo, Waltham, MA, USA) according to the manufacturer’s protocol, and reverse-transcribed into cDNA using MonScriptTM 5× RTIII All-in-One Mix (Monad Biotech Co., Ltd., Wuhan, Hubei, China). For qRT-PCR, a 20 μL reaction mixture was prepared with 10 μL of 2× AceQ Universal SYBR qRT-PCR Master Mix (Vazyme Biotech Co., Ltd., Nanjing, Jiangsu, China), 4 μM of forward primer, 4 μM of reverse primer and 400 ng cDNA. The reaction was performed in the Bio-Rad CFX96 QPCR System (Bio-Rad, Hercules, CA, USA). The primer sequences are listed in Table 5.

### 4.11. In Vivo Assessment

The experimental protocols were performed in accordance with the institutional guidelines for the care and use of laboratory animals at the Institute of Materia Medica, Chinese Academy of Medical Science and Peking Union Medical College. Seven to eight-week-old female BALB/c-nu nude mice were bred at Charles River (Charles River Laboratories, Beijing, China), certificate no: SYXK (Beijing, China) 2019-0023. The mice were inoculated with GH3 cells (1 × 10^6^ cells per mouse in 0.2 mL) and then treated with 20, 50 and 100 mg/kg TAM or 30 mg/kg TMZ (MCE, Shanghai, China) every day for 21 days. Body weight and tumor dimensions were measured every 3 days, and the tumor volumes was calculated as π/6× large diameter× (small diameter). On day 21, the mice were euthanized, and their blood was drawn. The tumor tissues were harvested, weighted and photographed. TMZ was selected as the positive control drug since it is effective against refractory PAs, including atypical adenomas and invasive adenomas [5,53].

### 4.12. Immunohistochemistry, TUNEL Assay and Imaging

The animals were anesthetized and perfused with 0.1 M phosphate buffered saline and 4% paraformaldehyde. The fixed tumor tissues were extracted, embedded in paraffin, and cut into 5 μm sections. The sections were subjected to IHC or TUNEL staining, counterstained with 4’,6-diamidino-2-phenylindole (DAPI), and observed under a fluorescence microscope (Nikon, Kyoto, Japan). 

### 4.13. Statistical Analysis

Data were expressed as mean ± SEM. Unpaired Student *t*-test or ANOVA was used to compare groups after confirming normal distribution and variance homogeneity. GraphPad software was used for statistical analysis, and *p* < 0.05 was considered statistically significant. 

## 5. Conclusions

TAM inhibits PA progression by inducing apoptosis and expression changes of apoptosis-related proteins, and reprogramming tumor-associated macrophages to the M1 phenotype via STAT6 inactivation and SHP1/SHP2 blockade.

## Figures and Tables

**Table 1:** GO analysis of DEGs associated with PAs.

a, BP			
GO Term	Function	Count	*p*-Value
GO:0042445	Hormone metabolic process	6	9.61 × 10^−5^
GO:0010817	Regulation of hormone levels	6	5 × 10^−4^
GO:0045893	Regulation of transcription, DNA-dependent	8	0.004535
GO:0051254	Positive regulation of RNA metabolic process	8	0.004745
GO:0009725	Response to hormone stimulus	7	0.005137
GO:0003006	Reproductive developmental process	6	0.005603
GO:0009719	Response to endogenous stimulus	7	0.008202
GO:0045665	Negative regulation of neuron differentiation	3	0.009053
GO:0031667	Response to nutrient levels	5	0.010641
GO:0045941	Positive regulation of transcription	8	0.011004
GO:0060541	Respiratory system development	4	0.011637
GO:0021510	Spinal cord development	3	0.012494
GO:0010033	Response to organic substance	9	0.012515
GO:0010628	Positive regulation of gene expression	8	0.012807
GO:0006357	Regulation of transcription from RNA polymerase	9	0.013109
GO:0001503	Ossification	4	0.013776
GO:0035295	Tube development	5	0.015426
GO:0009991	Response to extracellular stimulus	5	0.015426
GO:0060348	Bone development	4	0.016477
GO:0048608	Reproductive structure development	4	0.017561
GO:0045935	Regulation of nucleic acid metabolic process	8	0.018314
GO:0002684	Positive regulation of immune system process	5	0.01999
GO:0048732	Gland development	4	0.021049
GO:0051173	Regulation of nitrogen compound metabolic	8	0.021377
GO:0010001	Glial cell differentiation	3	0.022349
GO:0045944	Regulation of transcription from RNA polymerase	6	0.022552
GO:0010557	Regulation of macromolecule biosynthetic	8	0.023036
GO:0006590	Thyroid hormone generation	2	0.025889
GO:0007548	Sex differentiation	4	0.028124
GO:0031328	Positive regulation of cellular biosynthetic	8	0.028748
GO:0009891	Positive regulation of biosynthetic process	8	0.03078
GO:0010558	Regulation of macromolecule biosynthetic	7	0.031329
GO:0048754	Branching morphogenesis of a tube	3	0.032633
GO:0042063	Gliogenesis	3	0.032633
GO:0031327	Negative regulation of cellular biosynthetic	7	0.034828
GO:0035270	Endocrine system development	3	0.036402
GO:0009890	Negative regulation of biosynthetic process	7	0.038022
GO:0001763	Morphogenesis of a branching structure	3	0.041338
GO:0030182	Neuron differentiation	6	0.041736
GO:0006766	ViTamoxifenin metabolic process	3	0.042354
GO:0030522	Intracellular receptor-mediated signaling pathway	3	0.042354
GO:0048598	Embryonic morphogenesis	5	0.044701
GO:0030278	Regulation of ossification	3	0.045457
GO:0040007	Growth	4	0.0456
GO:0034097	Response to cytokine stimulus	3	0.04651
GO:0046457	Prostanoid biosynthetic process	2	0.046959
GO:0001516	Prostaglandin biosynthetic process	2	0.046959
GO:0016481	Negative regulation of transcription	6	0.049316
b, CC			
**GO term**	**Function**	**Count**	***p*-value**
GO:0044421	Extracellular region part	16	5.93 × 10^−5^
GO:0005576	Extracellular region	22	5.27 × 10^−4^
GO:0005578	Proteinaceous extracellular matrix	8	0.001055
GO:0031012	Extracellular matrix	8	0.001628
GO:0005615	Extracellular space	10	0.006775
c, MF			
**GO term**	**Function**	**Count**	***p*-value**
GO:0005201	Extracellular matrix structural constituent	4	0.006984
GO:0005506	Iron ion binding	6	0.012809
GO:0005509	Calcium ion binding	10	0.022046

GO: gene ontology; DEGs: differentially expressed genes; PAs: pituitary adenomas; BP: biological process; MF: molecular function; CC: cellular component.

**Table 2:** Core module analysis of the PPI network.

Module	Function Description	*p*-Value	Nodes	Genes
1	Negative regulation of response to stimulus	0.007499	4	*LEP*, *PTGS2*, *STAT6*, *ITPKB*
	T cell differentiation	0.00243	4	
2	Angiogenesis	0.016832	6	*ANG*, *FBLN1*, *MFAP5*, *EFEMP1*, *DCN*, *CXCL12*

PPI: protein–protein interaction; Corrected *p*-value < 0.05.

**Table 3:** The -Docking energy between TAM and STAT6 protein.

Gene	RMSD	-Docking Energy _original_	-Docking Energy _TAM_
STAT6	0.3199	14.3921	6.93868

RMSD: The root-mean-square deviation between the molecular conformations of the docked Ligand1 or Ligand2 and the initial conformations in the crystal structure of STAT6. -Docking energy _original_: The negative docking energy between original ligand and STAT6. -Docking energy _TAM_: The negative docking energy between TAM and STATTAM: tamoxifen.

**Table 4:** Details of pituitary adenomas data in GEO.

Sequence Number of Chips	GSE93825
Platform	GPL18281
Sample type	Pituitary human tissues
Sample	MACs and MICs
Reference	Cassarino et al. (2018)

GEO: Gene Expression Omnibus; MACs: macroadenomas; MICs: microadenomas.

**Table 5:** Primer sequences for qRT-PCR.

Genes	Primer Sequence (5′→3′)
*β-Actin*	Forward primer: TCTGTGTGGATTGGTGGCTCTA
	Reverse primer: CTGCTTGCTGATCCACATCTG
*Mrc-1*	Forward primer: GACTGCTGCTGAGTCCAGTT
	Reverse primer: AGGGATCGCCTGTTTTCCAG
*Arg-1*	Forward primer: ACATTGGCTTGCGAGACGTA
	Reverse primer: ATCACCTTGCCAATCCCCAG
*Ym1*	Forward primer: GGGCCCTTATTGAGAGGAGC
	Reverse primer: CCAGCTGGTACAGCAGACAA
*Fizz1*	Forward primer: CCTGCTGGGATGACTGCTAC
	Reverse primer: CAGTGGTCCAGTCAACGAGT
*IL-10*	Forward primer: CCAAGGTGTCTACAAGGCCA
	Reverse primer: GCTCTGTCTAGGTCCTGGAGT
*IL-6*	Forward primer: CCGGAGAGGAGACTTCACAG
	Reverse primer: CAGAATTGCCATTGCACAAC
*TNF-α*	Forward primer: AGCCGATGGGTTGTACCT
	Reverse primer: TGAGTTGGTCCCCCTTC
*INOS*	Forward primer: CTCTACAACATCCTGGAGCAAGTG
	Reverse primer: ACTATGGAGCACAGCCACATTGA

## Data Availability

Data presented in this study are included within the article.

## Supplementary Materials

The following are available online at https://www.mdpi.com/article/10.3390/ijms23052664/s1.
