# Supplementary material for: Tamoxifen Exerts Anticancer Effects on Pituitary Adenoma Progression via Inducing Cell Apoptosis and Inhibiting Cell Migration
Source: Int J Mol Sci. 2022 Feb 28;23(5):2664. doi: 10.3390/ijms23052664 (PMC8910631; doi:10.3390/ijms23052664)
Supplement: Supplementary file 1 [file ijms-23-02664-s001.zip › ijms-1616496-supplementary.pdf]

Supplementary Material

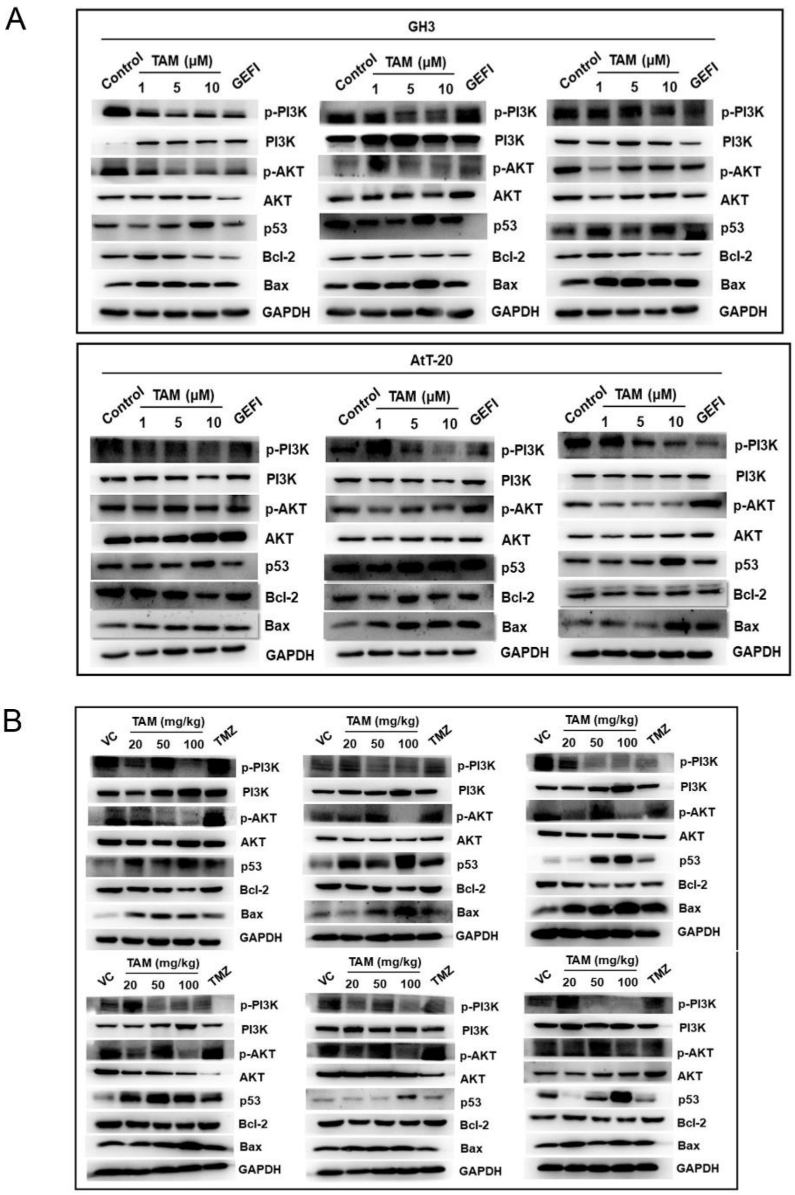

Supplementary Figure S1. TAM induced the apoptosis of PAs via the regulation of PI3K/AKT signaling pathway. A. TAM dose-dependently induced the apoptosis of PAs cells via PI3K/AKT signaling pathway. B. TAM dose-dependently induced the apoptosis of PAs via PI3K/AKT signaling pathway. TAM: tamoxifen; PAs: pituitary adenomas.

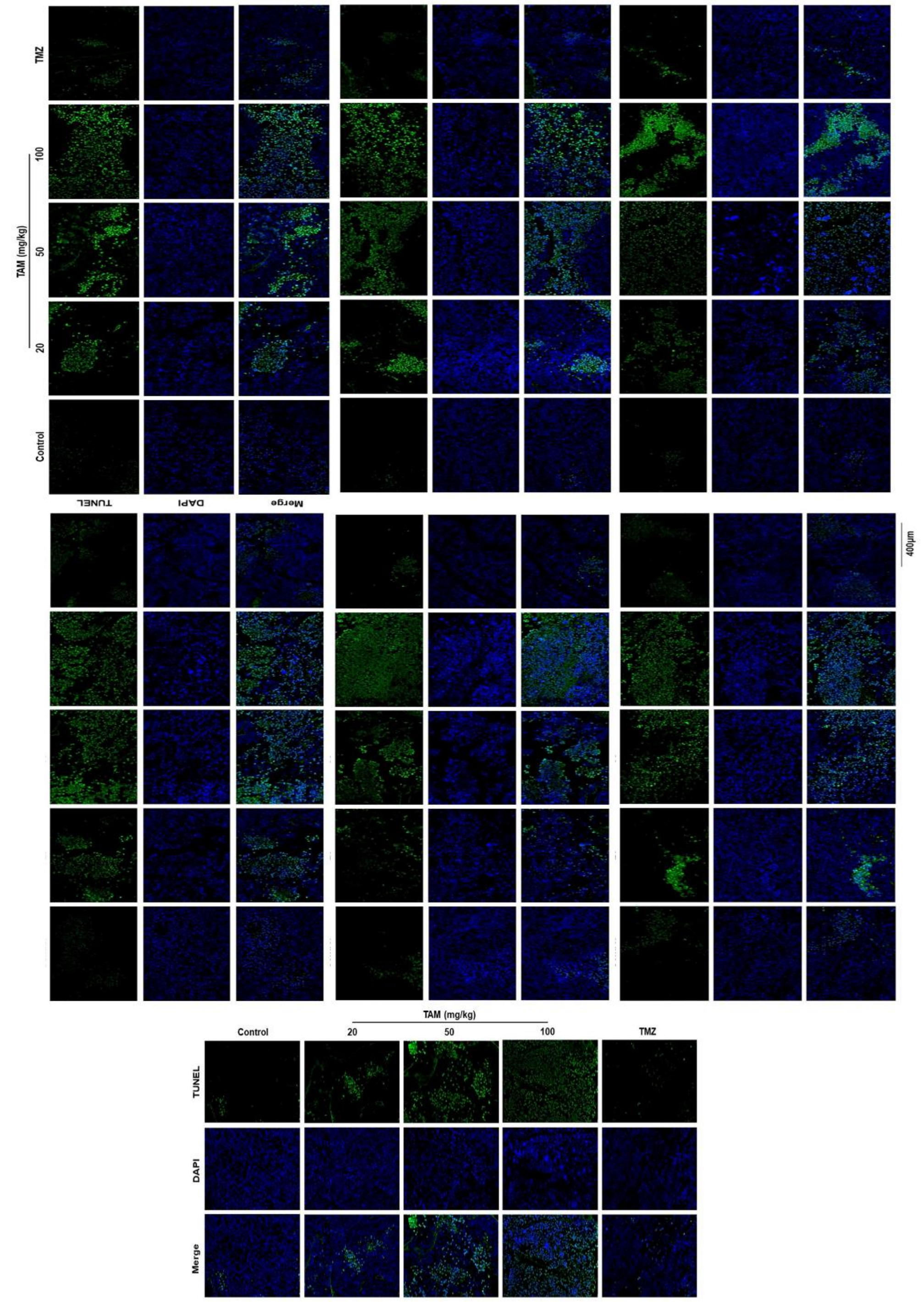

Supplementary Figure S2. TAM induced the apoptosis of PAs. Representative tumor tissues sections with TUNEL staining of apoptotic cells (Green). Cell nuclei were stained with DAPI (blue). Images are at 40× magnification. TAM: tamoxifen; TMZ: temozolomide.

A

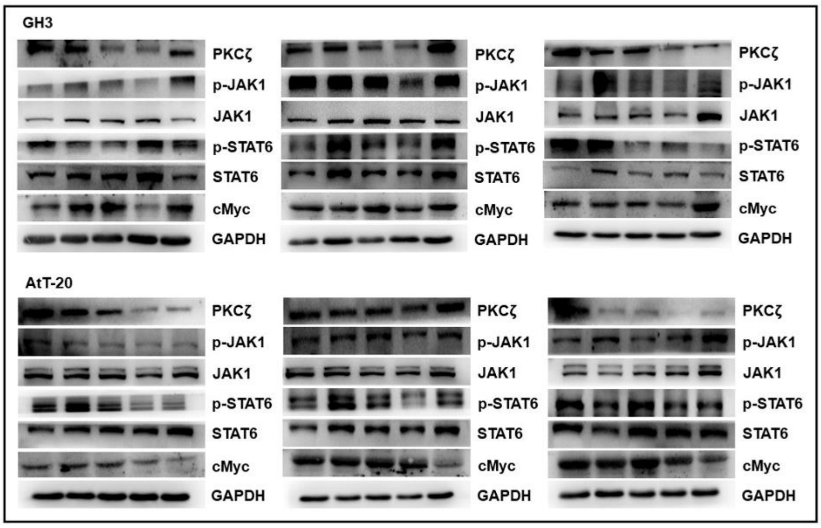

B

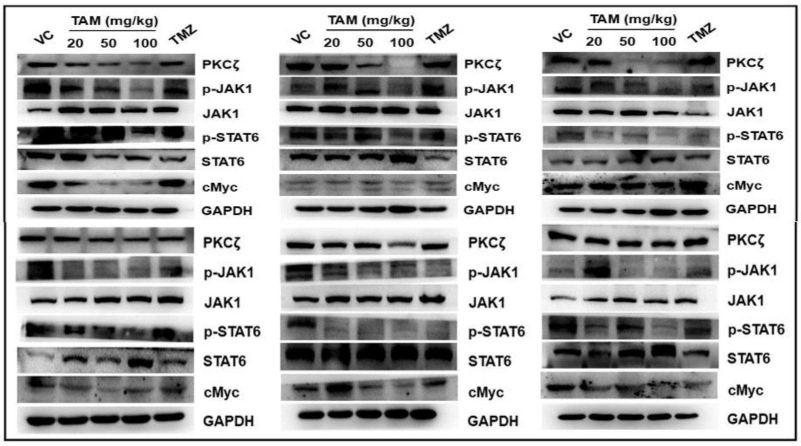

Supplementary Figure S3. TAM inactivated the JAK1/STAT6 signaling pathway in PAs. A. TAM inactivated the JAK1/STAT6 signaling pathway in GH3 and AtT-20 cells. B. TAM inactivated the JAK1/STAT6 signaling pathway in tumor tissues. TAM: tamoxifen; PAs: pituitary adenomas; TMZ: temozolomide.

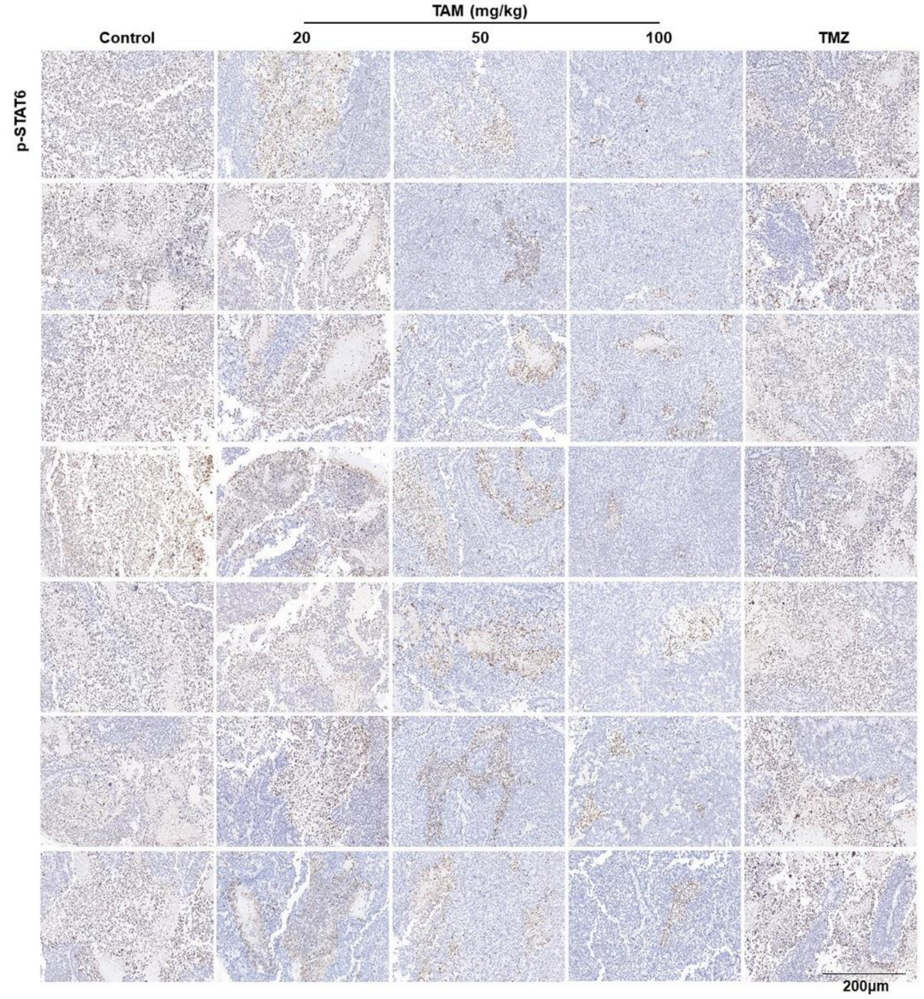

Supplementary Figure S4. TAM inhibited the activity of STAT6 in tumor tissues. Immunohistochemical (IHC) staining of p-STAT6 in representative tumor tissues sections. Images are at 20× magnification. TAM: tamoxifen; TMZ: temozolomide.

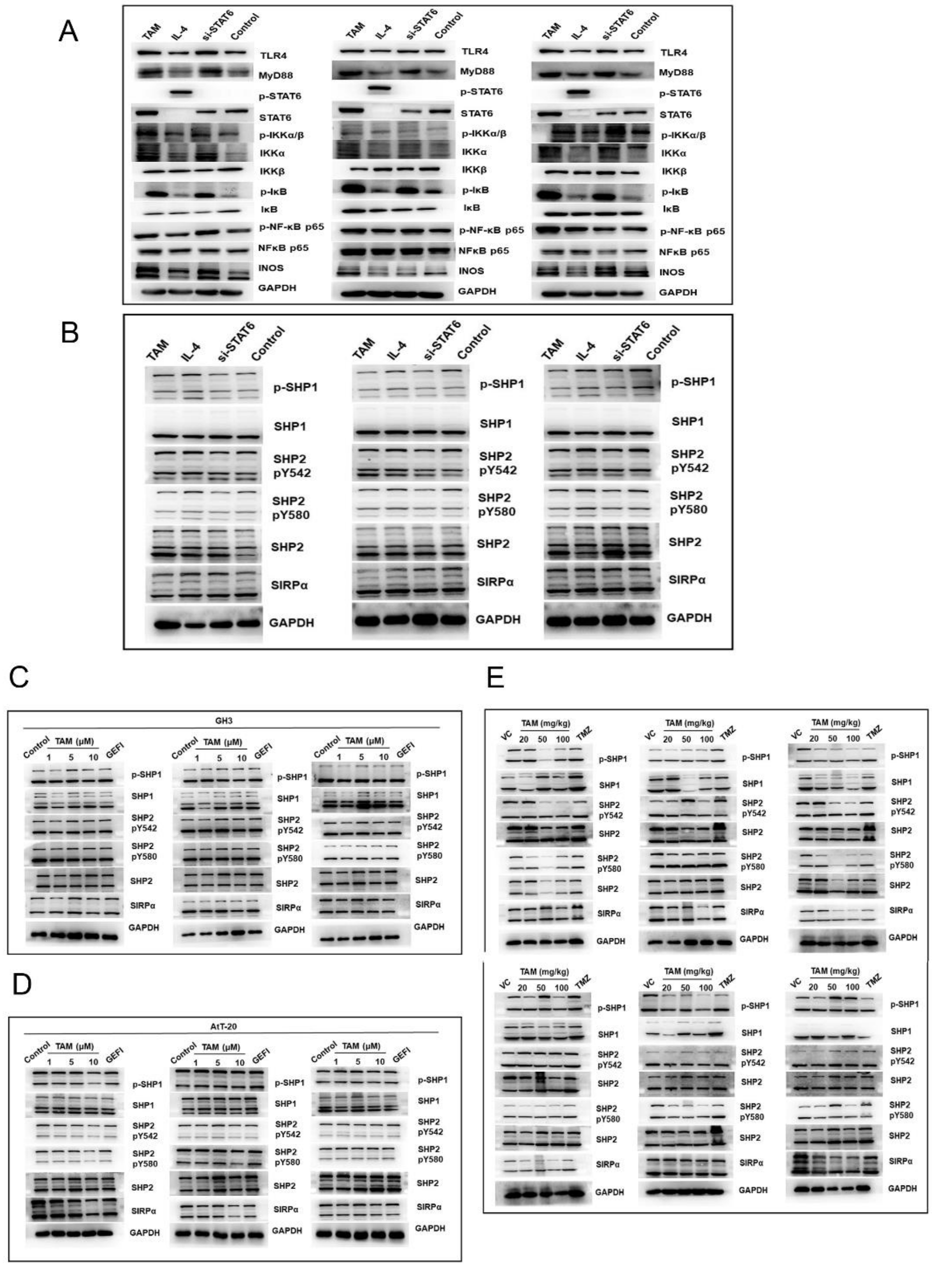

Supplementary Figure S5. A. TAM activated TLR4 signaling pathway induced by STAT6-inactivation. B. TAM inactivated immune checkpoint via increased TLR4 expression in RAW 264.7 cells. C. TAM inactivated immune checkpoint in GH3 cells. D. TAM inactivated immune checkpoint in AtT-20 cells. E. TAM inactivated immune checkpoint in pituitary adenomas tissues. TAM: tamoxifen; PAs: pituitary adenomas; GEFI: gefitinib; TMZ: temozolomide.

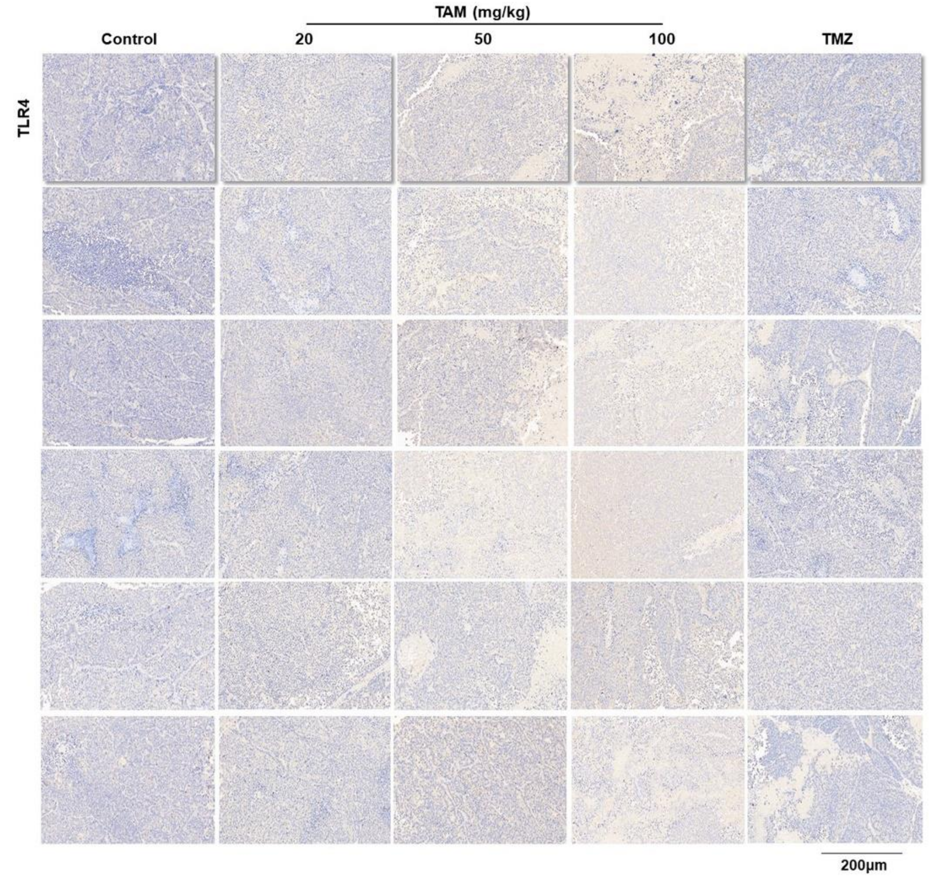

Supplementary Figure S6. TAM treatment markedly increased the number of Toll-like receptors 4 (TLR4+) macrophages in the tumor tissues. IHC staining of TLR4+ macrophages in representative tumor tissues sections. Images are at 20× magnification. VC vehicle control, refers to tumor-bearing mice treated with the vehicle. TAM: tamoxifen.

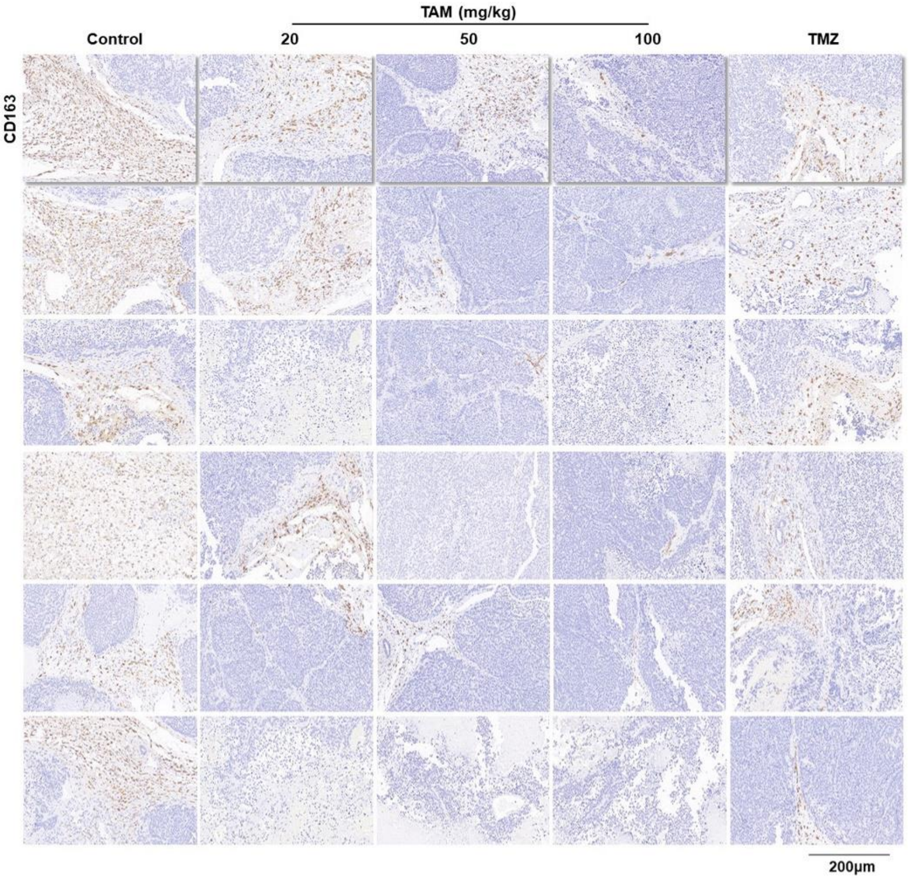

Supplementary Figure S7. TAM treatment markedly decreased the number of CD163+ macrophages in the tumor tissues. IHC staining of CD163+ macrophages in representative tumor tissues sections. Images are at 20× magnification. VC vehicle control, refers to tumor-bearing mice treated with the vehicle. TAM: tamoxifen.

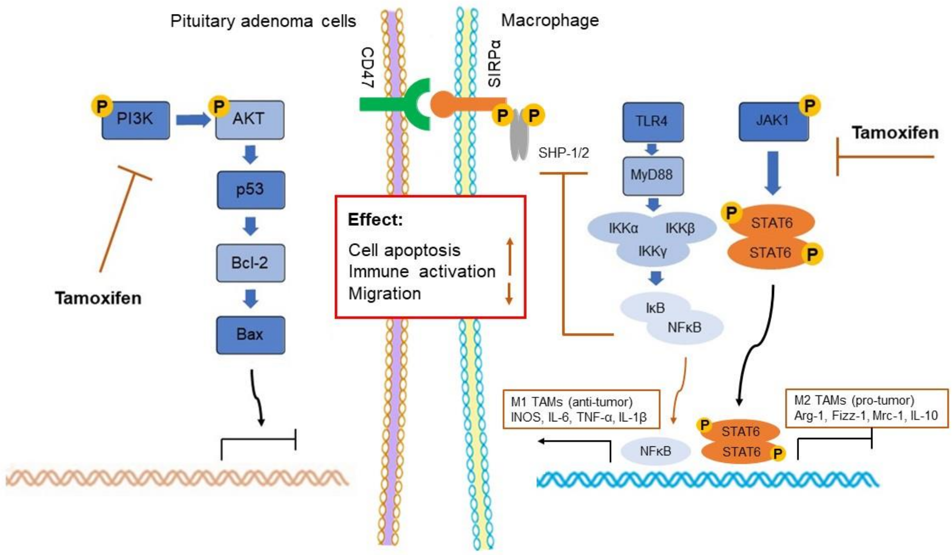

Supplementary Figure S8. The speculative schematic diagram of signaling pathway for the regulation of PAs growth by TAM. On the one hand, TAM inhibits PAs growth by inducing apoptosis of PAs cells. In PAs cells, TAM inhibits the expression of anti-apoptotic protein Bcl-2 and activates the expressions of pro-apoptotic proteins p53 and Bax through the inactivation of PI3K and AKT signals, thus promoting the apoptosis of PAs cells. On the other hand, TAM regulates the migration of PAs cells through selective activation of macrophages. In macrophages, TAM inhibits the polarization of tumor-associated macrophages (TAMs) to the M2 phenotype via the STAT6 signaling inactivation, and re-activates the TLR4/NF-κB signaling pathway, therefore reprogramming macrophages to an M1-profile, followed by decreasing the levels of macrophage-specific immune checkpoint SIRPα, and the down-regulation of phosphorous-SHP1/SHP2 and by the speculative inhibition of SIRPα and CD47 binding. In PAs cells, TAM may sequester of the binding of CD47 and SIRPα, followed by the transition from immune escape to activation, and inhibition of PAs cells migration. PAs: pituitary adenomas; TAM: tamoxifen; TAMs: tumor-associated macrophages.

Supplementary Table S1 Abbreviations and Acronyms

**Mots / Words**	**Abr. / Abrr.**
Pituitary adenomas	PAs
Food and Drug Administration	FDA
Tamoxifen	TAM
Phosphatidylinositol-3-kinase	PI3K
Protein kinase B	AKT
Gefitinib	GEFI
Signal transducer and activator of transcription 6	STAT6
Src-homology domain 2	SH2
SH-2 containing protein tyrosine phosphatase	SHP

Macroadenomas	MACs
Microadenomas	MICs
Gene Expression Omnibus	GEO
Differentially expressed genes	DEGs
Database for Annotation, Visualization and Integrated Discovery	DAVID
Protein-protein interaction	PPI
Leptin	LEP
Prostaglandin-endoperoxide synthase 2	PTGS2
C-X-C motif chemokine ligand 12	CXCL12
Inositol-trisphosphate 3-kinase B	ITPKB
Gene ontology	GO
Biologic process	BP
Cellular component	CC
Molecular function	MF
Growth hormone	GH
Adrenocorticotropic hormone	ACTH
Temozolomide	TMZ
B-cell lymphoma-2	Bcl-2
Bcl-2 associated X protein	Bax
Janus kinase 1	JAK1
Immuno histology chemistry	IHC
Mannose receptor C type-1	Mrc-1
Chil3	Ym-1
Retnla	Fizz-1
Arginase-1	Arg-1
Inducible Nitric Oxide Synthase	INOS
Toll-like receptors 4	TLR4
Myeloid differentiation factor 88	MyD88
Ikappa B kinase	IKK
Inhibitor of NF-κB	IκB
Nuclear factor κB	NF-κB
Signal regulatory proteins α	SIRPα
Search Tool for the Retrieval of Interacting Genes	STRING
Molecular Complex Detection	MCODE
Protein data bank	PDB
American Type Culture Collection	ATCC
Dulbecco’s modified Eagle’s medium	DMEM
Fetal bovine serum	FBS

Enzyme-linked immunosorbent assay	ELISA
Epidermal growth factor receptor	EGFR
Small interfering RNA	siRNA
Radio immunoprecipitation assay	RIPA
4',6-diamidino-2-phenylindole	DAPI
